# Clinical Correlates of the NR3C1 Gene Methylation at Various Stages of Psychosis

**DOI:** 10.1093/ijnp/pyaa094

**Published:** 2020-12-07

**Authors:** Błażej Misiak, Jerzy Samochowiec, Anna Konopka, Barbara Gawrońska-Szklarz, Jan Aleksander Beszłej, Elżbieta Szmida, Paweł Karpiński

**Affiliations:** 1 Department of Psychiatry, Wroclaw Medical University, Wroclaw, Poland; 2 Department of Psychiatry, Pomeranian Medical University, Szczecin, Poland; 3 Department of Pharmacokinetics and Therapeutic Drug Monitoring, Pomeranian Medical University, Szczecin, Poland; 4 Independent Clinical Psychology Unit, Department of Psychiatry, Pomeranian Medical University, Szczecin, Poland; 5 Department of Genetics, Wroclaw Medical University, Wroclaw, Poland; 6 Laboratory of Genomics & Bioinformatics, Institute of Immunology and Experimental Therapy, Polish Academy of Sciences, Wroclaw, Poland

**Keywords:** Childhood trauma, cortisol, epigenetics, psychotic disorder, stress

## Abstract

**Background:**

Dysregulation of epigenetic processes might account for alterations of the hypothalamic-pituitary-adrenal axis observed in patients with schizophrenia. Therefore, in this study, we aimed to investigate methylation of the glucocorticoid receptor (*NR3C1*) gene in patients with schizophrenia-spectrum disorders, individuals at familial high risk of schizophrenia (FHR-P), and healthy controls with respect to clinical manifestation and a history of psychosocial stressors.

**Methods:**

We recruited 40 first-episode psychosis patients, 45 acutely relapsed schizophrenia (SCZ-AR) patients, 39 FHR-P individuals, and 56 healthy controls. The level of methylation at 9 CpG sites of the *NR3C1* gene was determined using pyrosequencing.

**Results:**

The level of *NR3C1* methylation was significantly lower in first-episode psychosis patients and significantly higher in SCZ-AR patients compared with other subgroups of participants. Individuals with FHR-P and healthy controls had similar levels of *NR3C1* methylation. A history of adverse childhood experiences was associated with significantly lower *NR3C1* methylation in all subgroups of participants. Higher methylation of the *NR3C1* gene was related to worse performance of attention and immediate memory as well as lower level of general functioning in patients with psychosis.

**Conclusions:**

Patients with schizophrenia-spectrum disorders show altered levels of *NR3C1* methylation that are significantly lower in first-episode psychosis patients and significantly higher in SCZ-AR patients. Higher methylation of the *NR3C1* gene might be related to cognitive impairment observed in this clinical population. The association between a history of adverse childhood experiences and lower *NR3C1* methylation is not specific to patients with psychosis. Longitudinal studies are needed to establish causal mechanisms underlying these observations.

**Significance Statement**

Dysregulation of the hypothalamic-pituitary-adrenal (HPA) axis activity has been reported in patients with schizophrenia and might progress over time. Epigenetic processes, linking exposure to environmental factors with gene expression, may impact the HPA axis activity. Therefore, we investigated the level of methylation of the glucocorticoid receptor (*NR3C1*) gene in patients with schizophrenia-spectrum disorders at various stages of illness. The level of *NR3C1* methylation was significantly lower in patients with first-episode psychosis and significantly higher in acutely relapsed schizophrenia patients compared with controls. No significant changes in the level of *NR3C1* methylation were found in unaffected offspring of schizophrenia patients. Higher level of *NR3C1* methylation was related to worse cognitive performance in patients with psychosis. A history of adverse childhood experiences was associated with lower *NR3C1* methylation. These findings suggest that methylation of the *NR3C1* gene can increase over time, contributing to cognitive decline in patients with schizophrenia.

## Introduction

Psychotic disorders are complex phenotypes with a variety of genetic and environmental factors involved in their pathophysiology. There is now a general consensus that the genetic background of psychosis is related to the interplay of multiple risk variants with small effect size estimates (Misiak et al., 2016, 2017). In turn, variants with high effect size estimates are rarely reported in this group of patients (Zhuo et al., 2017). Additionally, several environmental factors have been found to increase a risk of psychosis and include perinatal complications and infections, adverse childhood experiences (ACEs), adult life stressors as well as substance use (Davis et al., 2016).

Following this etiological complexity, it has been proposed that epigenetic processes may improve the understanding of psychosis pathophysiology. The term “epigenetics” refers to various modifications that impact the expression of genes without altering their sequence. These processes include DNA methylation and hydroxymethylation, histone modifications, and microRNA signaling (Jaenisch and Bird, 2003). Notably, epigenetic processes can be influenced by various environmental exposures, such as nutritional deficiencies, substance use, infections, or psychosocial stressors (Feil and Fraga, 2012; Richetto and Meyer, 2020). It has been found that schizophrenia and other psychotic disorders can be associated with impaired epigenetic regulation (Smigielski et al., 2020). For instance, altered DNA methylation profiles have been observed in various tissues in patients with schizophrenia. Moreover, concordant patterns of altered DNA methylation between peripheral blood leukocytes and various brain regions have been demonstrated in patients with schizophrenia (Van Den Oord et al., 2016; Lin et al., 2018).

It has been reported that psychosocial stress, especially ACEs, can impact DNA methylation (Tomassi and Tosato, 2017). For instance, there is evidence that childhood maltreatment may alter the hypothalamic-pituitary-adrenal (HPA) axis activity via methylation of the glucocorticoid receptor (*NR3C1*) gene (McGowan et al., 2009; Radtke et al., 2015; Shields et al., 2016). These findings have also been reported in patients with schizophrenia (Barker et al., 2020). Moreover, it has recently been found that patients with schizophrenia may have a different profile of *NR3C1* methylation compared with healthy controls (Liu et al., 2020). However, clinical correlates of *NR3C1* methylation were not tested in this study. In addition, significant differences in the level of *NR3C1* expression between patients with schizophrenia or schizoaffective disorder and healthy controls have not been confirmed (Lee et al., 2019). Interestingly, higher expression of the *NR3C1* gene has been observed in at-risk individuals converting to overt psychosis compared with non-converters (Iftimovici et al., 2020).

There is evidence that the HPA axis activity can change during the course of psychotic disorders (Labad, 2019). On the basis of a meta-analysis, Girshkin et al. (2014) revealed higher morning cortisol levels in patients with an established diagnosis of schizophrenia compared with first-episode psychosis (FEP) patients. Another meta-analysis demonstrated flattened cortisol awakening response in patients with schizophrenia and FEP but not in individuals at clinical high risk of psychosis (Berger et al., 2016). However, methylation of the *NR3C1* gene at various stages of psychosis has not been investigated so far. Taking into account these research gaps, we aimed to investigate the levels of *NR3C1* methylation in acutely relapsed schizophrenia patients (SCZ-AR), FEP patients, individuals at familial high risk of psychosis (FHR-P), and healthy controls. Additionally, we tested the relationship between clinical manifestation, psychosocial stress at various life periods, and the *NR3C1* methylation in these groups of participants.

## Methods

### Participants

Participants were represented by 40 FEP patients, 45 SCZ-AR patients, 39 FHR-P individuals, and 56 healthy controls. They were enrolled at 2 clinical sites in Poland, including the Department of Psychiatry at Wroclaw Medical University and Pomeranian Medical University in Szczecin. Patients were diagnosed according to the DSM-IV criteria using the Operational Criteria for Psychotic Illness checklist (McGuffin et al., 1991). The following diagnostic categories were established in FEP patients: schizophrenia (n = 14), schizoaffective disorder (n = 5), schizophreniform disorder (n = 7), brief psychotic disorder (n = 13), and delusional disorder (n = 1). The rationale underlying the inclusion of a broad FEP spectrum was based on the observation that this diagnostic construct is related to multisystemic biological dysregulations (Pillinger et al., 2019). The majority of patients with FEP were medicated on the day of recruitment with treatment duration up to 30 days. There were 2 antipsychotic-naïve patients. The dosage of antipsychotics on the day of recruitment was converted to chlorpromazine equivalents (CPZeq). Individuals with FHR-P included unaffected offspring of patients with schizophrenia. They were not consanguine with other participants of this study and had no history of psychiatric or psychological treatment attempts. In turn, healthy controls were recruited through advertisements. They had a negative history of mood and psychotic disorders in first- and second-degree relatives. All participants gave written informed consent, and the protocol of this study was approved by the Ethics Committee of Wroclaw Medical University, Poland.

### Clinical Assessment

Symptomatic manifestation was recorded using the following measures: (1) the Positive and Negative Syndrome Scale (PANSS) (Kay et al., 1987); (2) the Montgomery-Åsberg Depression Rating Scale (MADRS) (Montgomery and Åsberg, 1979); (3) the Young Mania Rating Scale (Young et al., 2011), and (4) the Global Assessment of Functioning (GAF) (Hall, 1995). The Repeatable Battery for the Assessment of Neuropsychological Status (RBANS) was used to record cognitive performance (Randolph et al., 1998). The RBANS scores 5 domains of cognitive performance using 12 tasks: (1) immediate memory—list learning and story memory; (2) visuospatial/constructional functions—figure copy and line orientation; (3) language—picture naming and semantic fluency; (4) attention—digit span and coding; and (5) delayed memory—list recall, list recognition, story memory, and figure recall.

### Measures of Psychosocial Stress

A history of ACEs was recorded using the Childhood Experience of Care and Abuse Questionnaire (Bifulco et al., 2005). This self-report was administered to assess the following ACEs before the age of 17 years: parental loss, parental antipathy and neglect, physical abuse, and sexual abuse. The Childhood Experience of Care and Abuse Questionnaire has good psychometric properties and has been tested widely in patients with psychosis (Fisher et al., 2011).

Lifetime stressors were evaluated using the List of Threatening Experiences (Brugha and Cragg, 1990). The List of Threatening Experiences is a self-report used to record selected psychosocial stressors, including (1) serious illness, injury, or assault to self; (2) serious illness, injury, or assault to close relative; (3) death of parent, child, or spouse; (4) death of close friend or other relatives; (5) separation due marital problems; (6) broke off a steady relationship; (7) serious problems of close friend, neighbor, or relative; (8) becoming unemployed or seeking work >1 month; (9) being fired from job; (10) major financial crisis; (11) problems with police and court appearance; and (12) something valuable lost or stolen. The number of lifetime stressors, ranging between 0 and 12, was used in this study.

The Perceived Stress Scale was administered to assess intensity of proximal stress. The Perceived Stress Scale measures self-perception of stress over the preceding month. It is based on 10 questions rated on a 5-point Likert scale, ranging from 0 (never) to 4 (very often). The total score ranges between 0 and 40, with higher scores indicating higher levels of perceived stress.

### Cortisol Levels and Pyrosequencing

Fasting blood samples were collected between 7 am and 9 am. Serum levels of cortisol were measured using the electrochemiluminescence analysis (Cobas e411 analyzer, Roche, Switzerland). The Prepito DNA Blood250 Kit was used to obtain DNA from peripheral blood leukocytes. All clinical assessments were performed on the day of blood sampling. Similarly, the measures of stress were administered on the day of blood sampling.

**Figure 1.**
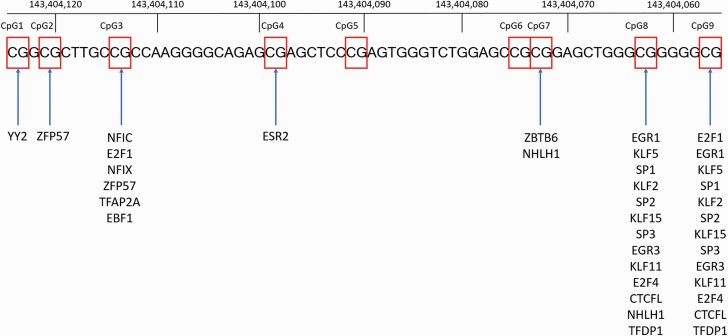
Location of CpG sites analyzed in the present study according to the Genome Reference Consortium Human Build 38 patch release 7 primary assembly. Selected CpG sites were marked with red boxes. Binding sites of transcription factors to the JASPAR database (version 8) were marked with blue arrows. Expression of NHLH1 has been detected only in the brain (predominantly in the cerebellum). Expression of other transcription factors has been observed in the brain and blood cells.

Nine CpG sites at the NR3C1 exon 1F, including CpG1 (hg38: 143404124), CpG2 (chr5: 143404121), CpG3 (chr5: 143404114), CpG4 (chr5: 143404099), CpG5 (chr5: 143404091), CpG6 (chr5: 143404075), CpG7 (chr5: 143404073), CpG8 (chr5: 143404063), and CpG9 (chr5: 143404057), were selected for pyrosequencing based on their proximity to binding sites of transcription factors (Figure 1). We assessed the sequence of the *NR3C1* exon 1F located at chr5:143404057-143404124 using the collection of 810 human transcription factor binding sites from the JASPAR database (version 8) (Fornes et al., 2020). Candidate transcription factors were identified by overlap of the promoter of exon 1F sequence with predicted motifs using a relative profile score threshold of 85%. Subsequently, all selected transcription factors were assessed for their tissue-specific transcription in the Human Protein Atlas (Uhlén et al., 2015). Transcription factors expressed in the blood and/or brain cells were presented in Figure 1.

Bisulfite treatment was performed using 1400 ng of sample genomic DNA and the EZ DNA Methylation-Direct kit (Zymo Research, Orange, CA). A total of 42 ng of bisulfite-modified DNA was used as a template in the polymerase chain reaction (PCR). The PCR was performed in a total volume of 50 µL for 35 cycles using the FastStart High-Fidelity Taq DNA Polymerase (1.0 U), MgCl_2_ solution (3.5 mM), dNTPs (0.2 mM), sense primer (0.24 µM), and antisense primer (0.18 µM) with denaturation at 95°C for 30 seconds, annealing for 45 seconds at 57°C and 53°C, and extension at 72°C for 1 minute. The following sets of primers were used: (1) sense primer: 5’-GTGTAATTTYGTAGTTTTTTTYGAAGTGATATATT-3’; (2) anti-sense primer: 5’-AACCACCRAATTTCTCCAATTTCTTTTCT-3’; and (3) sequencing primer: 5’- TTGTTATTYGTAGGGGTATTG-3’ (PCR product: 254 bp, annealing temperature: 62°C). The PCR product was electrophoresed on 1% agarose gel, stained with ethidium bromide, and visualized for appropriate and pure product before proceeding with all analyses using the Bio-Rad Laboratories (Hercules, CA) Gel-Doc UV illuminator. Methylation percentage of each CpG was measured using the Qiagen (Valencia, CA) Pyromark Q96 ID pyrosequencer and sequencing primers.

### Statistics

Due to multiple CpG sites tested in this study, principal component analysis was performed. The number of components extracted was based on the analysis of the scree plot (eigenvalues > 1). The direct oblimin method was used for factor rotation. Sampling adequacy and sphericity were tested using the Keiser-Mayer-Olkin measure and Bartlett’s test, respectively. Factor loadings in the pattern matrix >0.3 were considered contributing to the specific component. Mean methylation of CpG sites contributing to extracted components was included as the measure of the *NR3C1* methylation.

Between-group differences were tested using the Mann-Whitney U test or the Kruskal-Wallis test (continuous variables) and the chi-squared test (categorical variables). In case of significant results of the Kruskal-Wallis test, post-hoc comparisons with the Dunn-Bonferroni test were carried out. Bivariate correlations were assessed by analysis of the Spearman’s rank correlation coefficients.

The ANCOVA was further used to test differences in the level of *NR3C1* methylation. Similarly, significant bivariate correlations between the *NR3C1* methylation, stress measures, and symptomatic manifestation were tested using linear regression analysis. Covariates were selected based on the analysis of pairwise comparisons and bivariate correlations in the whole sample of participants. The following variables were considered as potential covariates: age, sex, body mass index (BMI), cigarette smoking status, presence of comorbid physical health impairments, use of medications for physical comorbidities, illness duration, CPZeq, and cortisol levels. The *NR3C1* methylation level was included as a dependent variable, and the group status and a history of specific ACEs represented independent variables.

Results were considered statistically significant if the *P* value was less than .05. The Statistical Package for Social Sciences version 20 (SPSS Inc., Chicago, IL) was used to perform data analysis.

## Results

General characteristics of the sample were presented in Table 1. There were significant between-group differences in terms of age, number of education years, BMI, cigarette smoking rates, and cognitive performance. Cortisol levels were significantly higher in FEP and SCZ-AR patients compared with healthy controls. Additionally, cortisol levels were significantly higher in SCZ-AR patients than in FHR-P individuals. Patients with SCZ-AR had significantly higher scores of negative symptoms, longer illness duration, greater CPZeq, and lower GAF scores compared with FEP patients. Notably, there were no significant between-group differences in the rates of somatic comorbidities and the use of non-psychiatric medications. Somatic comorbidities were as follows: allergies and asthma (FEP: n = 4, SCZ-AR: n = 0, FHR-P: n = 5, and healthy controls: n = 2), cardiovascular diseases (FEP: n = 0, SCZ-AR: n = 3, FHR-P: n = 2, and healthy controls: n = 1), type 2 diabetes (FEP: n = 0, SCZ-AR: n = 2, FHR-P: n = 0, and healthy controls: n = 1), and thyroid diseases (FEP: n = 1, SCZ-AR: n = 3, FHR-P: n = 3, and healthy controls: n = 0).

**Table 1.** General Characteristics of the Sample

	FEP		SCZ-AR		FHR-P		HCs		*P*	Post-hoc comparisons
	n	Mean ± SD, or n (%)	n	Mean ± SD, or n (%)	n	Mean ± SD, or n (%)	n	Mean ± SD, or n (%)		
Age, y	40	28.1 ± 7.3	45	45.2 ± 12.6	39	36.9 ± 11.2	56	32.5 ± 8.2	**<.001**	1 < 3, 1 < 2, 2 > 3, 2 > 4
Sex, males	40	20 (50.0)	45	25 (55.6)	39	14 (35.9)	56	22 (39.3)	.215	—
Education, y	40	13.6 ± 2.5	38	12.6 ± 3.0	37	15.5 ± 3.6	54	15.8 ± 2.5	**<.001**	1 < 4, 2 < 3, 2 < 4
BMI, kg/m^2^	40	23.7 ± 3.8	40	26.5 ± 5.1	37	24.5 ± 4.0	56	23.8 ± 3.5	**.013**	1 < 2, 2 > 4
Cigarette smoking	40	15 (37.5)	40	22 (55.0)	37	6 (16.2)	56	5 (8.9)	**<.001**	1 > 4, 1 > 3, 2 > 1, 2 > 4, 2 > 3, 3 > 4
Somatic comorbidities	40	6 (15.0)	45	7 (15.6)	39	4 (10.3)	56	11 (19.6)	.670	—
Non-psychiatric medications	40	2 (5.0)	45	7 (15.6)	39	3 (7.7)	56	8 (14.3)	.328	—
Parental loss	37	10 (27.0)	36	13 (36.1)	34	16 (47.1)	54	12 (22.2)	.084	—
Parental antipathy	37	10 (27.0)	36	18 (50.0)	34	12 (35.3)	54	16 (29.6)	.140	—
Parental neglect	37	6 (16.2)	36	13 (36.1)	34	12 (35.3)	54	14 (25.9)	.261	—
Physical abuse	37	13 (35.1)	36	17 (47.2)	34	12 (35.3)	54	13 (24.1)	.157	—
Sexual abuse	37	5 (13.5)	36	7 (19.4)	33	5 (15.1)	54	3 (5.6)	.239	—
Any ACE	37	24 (64.9)	36	28 (77.8)	34	27 (79.4)	54	31 (57.4)	.086	—
RBANS—immediate memory	40	42.7 ± 8.4	44	33.5 ± 11.3	37	49.6 ± 6.5	52	51.9 ± 6.0	**<.001**	1 > 2, 2 < 3, 1 < 3, 1 < 4, 2 < 4
RBANS—visuospatial/constructional abilites	40	34.7 ± 5.4	44	30.0 ± 8.2	37	36.7 ± 3.9	52	38.1 ± 2.2	**<.001**	1 > 2, 2 < 3, 1 < 4, 2 < 4
RBANS—language	40	28.2 ± 6.1	44	24.9 ± 6.6	37	32.7 ± 6.2	52	33.7 ± 6.5	**<.001**	2 < 3, 1 < 3, 1 < 4, 2 < 4
RBANS—attention	40	54.2 ± 12.2	44	35.6 ± 11.8	37	63.1 ± 13.6	52	68.9 ± 8.9	**<.001**	1 > 2, 2 < 3, 1 < 4, 2 < 4
RBANS—delayed memory	40	46.9 ± 7.7	44	39.0 ± 11.3	37	51.9 ± 5.5	52	56.0 ± 4.5	**<.001**	1 > 2, 2 < 3, 1 < 3, 1 < 4, 2 < 4
LTE	40	5.0 ± 2.5	41	6.8 ± 2.6	35	4.3 ± 1.9	54	3.6 ± 2.2	**<.001**	1 > 4, 2 > 4, 2 > 3, 2 > 1
PSS-10	40	23.7 ± 6.5	36	22.2 ± 6.3	36	23.6 ± 4.3	54	22.5 ± 4.0	.444	—
Illness duration, wk	40	43.8 ± 87.8	45	651.7 ± 526.9	—	—	—	—	**<.001**	—
PANSS-P	40	12.9 ± 5.2	40	15.2 ± 4.9	—	—	—	—	.053	—
PANSS-N	40	18.1 ± 8.4	40	23.8 ± 9.5	—	—	—	—	**.008**	—
MADRS	40	8.3 ± 8.1	38	7.8 ± 8.3	—	—	—	—	.743	—
YMRS	40	2.1 ± 5.1	38	2.1 ± 5.0	—	—	—	—	.758	—
GAF	40	54.2 ± 17.1	39	35.3 ± 14.0	—	—	—	—	**<.001**	—
CPZeq, mg/d	40	300.1 ± 169.7	37	467.7 ± 219.8	—	—	—	—	**.001**	—
Cortisol, nmol/L	40	338.6 ± 73.3	23	448.9 ± 151.9	37	327.9 ± 158.7	55	272.8 ± 87.2	**<.001**	1 > 4, 2 > 4, 2 > 3

Abbreviations: ACEs, adverse childhood experiences; BMI, body mass index; CPZeq, chlorpromazine equivalent dosage; FEP, first-episode psychosis; FHR-P, individuals at familial high risk of psychosis; GAF, the Global Assessment of Functioning; HCs, healthy controls; LTE, List of Threatening Experiences; MADRS, Montgomery- Åsberg Depression Rating Scale; PANSS-N, Positive and Negative Syndrome Scale (subscale of negative symptoms); PANSS-P, Positive and Negative Syndrome Scale (subscale of positive symptoms); PSS, Perceived Stress Scale; RBANS, Repeatable Battery for the Assessment of Neuropsychological Status; SCZ-AR, acutely relapsed schizophrenia patients; YMRS, Young Mania Rating Scale.

Significant differences (*P* < .05) are marked in bold.

The principal component analysis extracted 2 components (see supplementary Figure 1 for a scree plot and supplementary Table 1 for factor loadings). The same components were extracted when the analysis was limited to age-matched subgroups of participants. Component 1 included 5 CpG sites (CpG1, CpG3, CpG5, CpG6, and CpG9), and component 2 was based on 4 CpG sites (CpG2, CpG4, CpG7, and CpG8). The cumulative percentage of variance explained by both components was 49.042 in the whole sample (component 1: 28.708%, component 2: 20.335%). The Keiser-Mayer-Olkin measure was 0.683 and the results of Bartlett’s test of sphericity were significant (chi-square = 342.45, *P* < .001).

**Figure 2.**
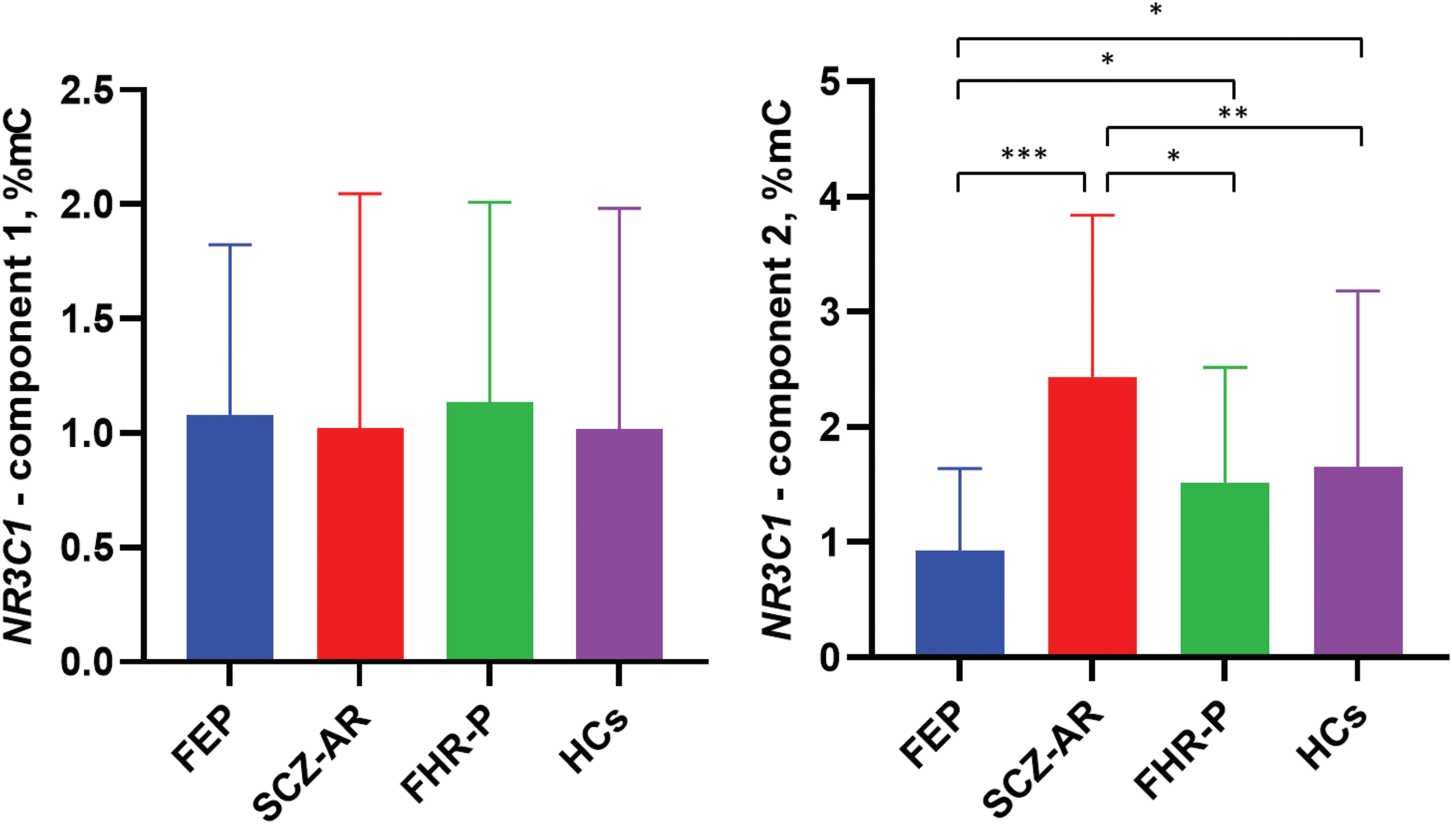
Mean levels of the *NR3C1* methylation. Error bars represent SD. *P < .05, **P < .01, ***P < .001. Abbreviations: FEP, first-episode psychosis; FHR-P, familial high risk of psychosis; HCs, healthy controls; SCZ-AR, acutely relapsed schizophrenia.

Patients with FEP had significantly lower methylation of component 2 compared with other subgroups of participants (Figure 2). In turn, SCZ-AR patients had significantly higher methylation of component 2 compared with other subgroups of participants. Individuals with FHR-P and healthy controls had similar levels of component 2 methylation. Analysis of single CpG sites included in the component 2 revealed significant between-group differences in the methylation of CpG2, CpG4, and CpG8 (supplementary Table 2). No significant between-group differences in methylation of component 1 were found. Similar results were obtained when the sample was limited to age-matched subgroups (supplementary Figure 2). However, patients with SCZ-AR had significantly lower methylation of CpG1 compared with FHR-P individuals and healthy controls. In turn, patients with FEP had significantly higher methylation of CpG5 compared with healthy controls (supplementary Table 2).

**Figure 3.**
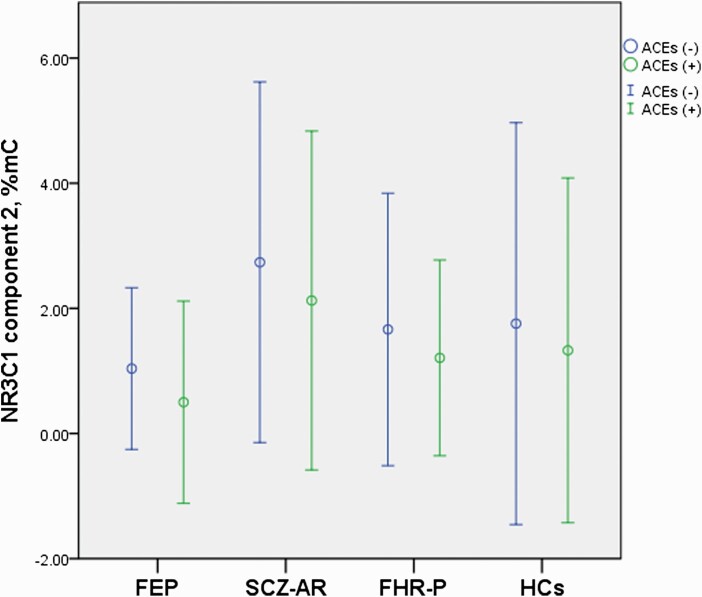
Mean methylation of the *NR3C1* component 2 with respect to a history of adverse childhood experiences. Error bars represent SD. Abbreviations: ACEs, adverse childhood experiences; FEP, first-episode psychosis; FHR-P, familial high risk of psychosis; HCs, healthy controls; SCZ-AR, acutely relapsed schizophrenia.

The association between potential confounding factors and methylation of the *NR3C1* components was presented in supplementary Table 3. Age (r = 0.209, *P* = .005), illness duration (r = 0.444, *P* < .001), and CPZeq (r = 0.266, *P* = .020) were related to significantly higher methylation of component 2. None of potential confounding factors was significantly associated with methylation of component 1. The ANCOVA demonstrated significant main effects of group (FEP vs SCZ-AR vs FHR-P vs healthy controls) on the level of component 2 methylation in all models (Table 2) after controlling for the effects of age, illness duration, and CPZeq. There was also a significant main effect of a history of any ACEs on the level of component 2 methylation. More specifically, participants with a history of any ACEs had significantly lower methylation of component 2 (Figure 3).

**Table 2.** ANCOVA Testing for the Effects of Diagnostic Group and ACEs on *NR3C1* Methylation

*NR3C1*, %mC	Independent variable	Parental loss	Parental antipathy	Parental neglect	Physical abuse	Sexual abuse	Any ACEs
Component 1	Group	F = 0.263, *P* = .852	F = 0.298, *P* = .827	F = 1.628, *P* = .185	F = 0.437, *P* = .727	F = 0.023, *P* = .995	F = 0.082, *P* = .970
	ACEs	F = 3.280, *P* = .072	F = 0.006, *P* = .939	F = 0.194, *P* = .660	F = 0.264, *P* = .608	F = 0.759, *P* = .385	F = 0.422, *P* = .517
	Group × ACEs	F = 0.968, *P* = .409	F = 0.494, *P* = .687	F = 0.210, *P* = .891	F = 1.162, *P* = .326	F = 0.215, *P* = .886	F = 2.021, *P* = .113
Component 2	Age	F = 0.004, *P* = .949	F = 0.016, *P* = .900	F = 0.055, *P* = .815	F = 0.012, *P* = .914	F = 0.045, *P* = .832	F = 0.005, *P* = .942
	Illness duration	F = 0.274, *P* = .602	F = 0.223, *P* = .637	F = 2.098, *P* = .150	F = 0.282, *P* = .596	F = 0.242, *P* = .624	F = 0.087, *P* = .769
	CPZeq	F = 1.058, *P* = .305	F = 1.102, *P* = .296	F = 1.179, *P* = .279	F = 0.429, *P* = .514	F = 0.792, *P* = .375	F = 0.943, *P* = .333
	Group	**F = 3.565, *P* = .016**	**F = 3.912, *P* = .010**	**F = 4.496, *P* = .005**	**F = 3.733, *P* = .013**	**F = 4.564, *P* = .004**	**F = 3.469, *P* = .018**
	ACEs	F = 0.566, *P* = .453	F = 3.537, *P* = .062	F = 0.075, *P* = .785	F = 0.337, *P* = .562	F = 0.521, *P* = .472	**F = 3.582, *P* = .015**
	Group × ACEs	F = 0.329, *P* = .804	F = 0.078, *P* = .972	F = 1.501, *P* = .217	F = 0.823, *P* = .483	F = 2.281, *P* = .082	F = 0.031, *P* = .993

Abbreviations: ACEs, adverse childhood experiences; CPZeq, chlorpromazine equivalent dosage.

Significant effects (*P* < .05) are marked in bold.

Bivariate correlations with symptomatic manifestation and other stress measures were presented in Table 3. Higher methylation of component 2 was associated with significantly lower scores of the GAF and 3 RBANS subscales (immediate memory, attention, and delayed memory) in patients with psychosis. However, the correlation between the *NR3C1* component 2 methylation and delayed memory was significant in FEP patients but not in SCZ-AR patients. These correlations were not significant in other groups of participants. Linear regression analyses revealed that correlations between the level of component 2 methylation and the GAF score as well as the scores of immediate memory and attention were significant in FEP and SCZ-AR patients after controlling for the effects of potential confounding factors.

**Table 3.** Correlations Between the Level of *NR3C1* Methylation and Clinical Variables

	FEP		SCZ-AR		FHR-P		HCs	
	Component 1	Component 2	Component 1	Component 2	Component 1	Component 2	Component 1	Component 2
PSS	r = 0.155, *P* = .339	r = 0.241, *P* = .135	r = −0.060, *P* = .728	r = −0.196, *P* = .252	r = 0.099, *P* = .565	r = 0.090, *P* = .602	r = −0.004, *P* = .978	r = 0.124, *P* = .373
LTE	r = 0.004, *P* = .979	r = −0.084, *P* = .604	r = 0.046, *P* = .774	r = −0.036, *P* = .823	r = −0.137, *P* = .433	r = 0.068, *P* = .697	r = 0.096, *P* = .490	r = 0.221, *P* = .108
RBANS—immediate memory	r = −0.107, *P* = .513	**r = −0.442, *P* = .004** ^***a***^	r = 0.091, *P* = .556	**r = −0.398, *P* = .008** ^***b***^	r = 0.041, *P* = .808	r = 0.181, *P* = .285	r = 0.111, *P* = .434	r = −0.155, *P* = .273
RBANS—visuospatial/ constructional abilites	r = 0.056, *P* = .730	r = −0.284, *P* = .076	r = −0.048, *P* = .755	r = −0.073, *P* = .638	r = 0.114, *P* = .502	r = 0.236, *P* = .160	r = 0.033, *P* = .816	r = 0.129, *P* = .363
RBANS—language	r = 0.116, *P* = .476	r = −0.206, *P* = .203	r = −0.185, *P* = .228	r = 0.096, *P* = .536	r = 0.159, *P* = .347	r = 0.107, *P* = .530	r = 0.043, *P* = .760	r = −0.070, *P* = .622
RBANS—attention	r = 0.083, *P* = .611	**r = −0.384, *P* = .014** ^***c***^	r = −0.169, *P* = .273	**r = −0.452, *P* = .002** ^***d***^	r = 0.097, *P* = .566	r = 0.154, *P* = .363	r = 0.016, *P* = .908	r = 0.010, *P* = .944
RBANS—delayed memory	r = −0.144, *P* = .375	**r = −0.367, *P* = .020** ^***e***^	r = 0.111, *P* = .475	r = −0.102, *P* = .509	r = 0.114, *P* = .502	r = −0.103, *P* = .543	r = −0.108, *P* = .444	r = −0.031, *P* = .825
GAF	r = −0.042, *P* = .798	**r = −0.387, *P* = .014** ^***f***^	r = −0.109, *P* = .508	**r = −0.324, *P* = .044** ^***g***^	—	—	—	—
PANSS-P	r = 0.010, *P* = .951	r = −0.107, *P* = .512	r = 0.033, *P* = .839	r = 0.053, *P* = .744	—	—	—	—
PANSS-N	r = −0.143, *P* = .378	r = −0.169, *P* = .297	r = 0.042, *P* = .839	r = 0.015, *P* = .928	—	—	—	—
MADRS	r = −0.112, *P* = .491	r = −0.165, *P* = .309	r = 0.267, *P* = .106	r = 0.090, *P* = .592	—	—	—	—
YMRS	r = 0.244, *P* = .129	r = 0.019, *P* = .905	r = −0.234, *P* = .157	r = −0.179, *P* = .282	—	—	—	—

Abbreviations: FHR-P, individuals at familial high risk of psychosis; GAF, Global Assessment of Functioning; HCs, healthy controls; LTE, List of Threatening Experiences; MADRS, Montgomery-Åsberg Depression Rating Scale; PANSS-N, Positive and Negative Syndrome Scale (subscale of negative symptoms); PANSS-P, Positive and Negative Syndrome Scale (subscale of positive symptoms); PSS, Perceived Stress Scale; RBANS, Repeatable Battery for the Assessment of Neuropsychological Status; YMRS, Young Mania Rating Scale.

^*a*^Linear regression analysis: component 2 (B = −0.649, t = −2.566, *P* = .012, VIF = 1.302); age (B = −0.08, t = −0.664, *P* = .509, VIF = 1.748); illness duration (B = −0.003, t = −1.006, *P* = .318, VIF = 1.898); CPZeq (B = −0.008, t = −1.408, *P* = .164, VIF = 1.217).

^*b*^Linear regression analysis: component 2 (B = −0.442, t = −3.111, *P* = .04, VIF = 1.051); age (B = −0.023, t = −0.138, *P* = .891, VIF = 1.370); illness duration (B = −0.002, t = −0.445, *P* = .661, VIF = 1.341); CPZeq (B = −0.011, t = −1.157, *P* = .259, VIF = 1.049).

^*c*^Linear regression analysis: component 2 (B = −5.521, t = −2.032, *P* = .049, VIF = 1.015); age (B = −0.173, t = −0.623, *P* = .537, VIF = 1.107); illness duration (B = −0.018, t = −0.770, *P* = .447, VIF = 1.083); CPZeq (B = −0.008, t = −0.645, *P* = .523, VIF = 1.082).

^*d*^Linear regression analysis: component 2 (B = −0.462, t = −2.679, *P* = .014, VIF = 1.130); age (B = −0.252, t = −1.620, *P* = .119, VIF = 1.370); illness duration (B = −0.005, t = −1.222, *P* = .234, VIF = 1.341); CPZeq (B = 0.002, t = 0.119, *P* = .119, VIF = 1.049).

^*e*^Linear regression analysis: component 2 (B = −0.806, t = −0.441, *P* = .662, VIF = 1.015); age (B = −0.060, t = −0.319, *P* = .751, VIF = 1.107); illness duration (B = 0.009, t = 0.601, *P* = .552, VIF = 1.083); CPZeq (B = 0.003, t = 0.398, *P* = .693, VIF = 1.082).

^*f*^Linear regression analysis: component 2 (B = −0.470, t = −2.041, *P* = .049, VIF = 1.151); age (B = 0.352, t = 0.894, *P* = .378, VIF = 1.107); illness duration (B = −0.059, t = −0.059, t = −1.816, *P* = .078); CPZeq (B = 0.009, t = 0.537, *P* = .595, VIF = 1.082).

^*g*^Linear regression analysis: component 2 (B = −4.353, t = −2.244, *P* = .035, VIF = 1.051); age (B = −0.169, t = −0.753, *P* = .459, VIF = 1.370); illness duration (B = 0.008, t = 1.455, *P* = .159, VIF = 1.341); CPZeq (B = −0.006, t = −0.451, *P* = .656, VIF = 1.370).

Significant bivariate correlations (*P* < .05) are marked in bold.

## Discussion

This study demonstrated significantly lower methylation of 4 CpG sites at the *NR3C1* gene in FEP patients compared with FHR-P individuals and healthy controls. Interestingly, methylation of these CpG sites was significantly higher in SCZ-AR patients compared with FHR-P and healthy controls. No significant differences in methylation of the *NR3C1* between FHR-P individuals and healthy controls were found. Altogether, these findings suggest that methylation of the *NR3C1* gene might increase with subsequent exacerbations of schizophrenia. These findings are in agreement with recent meta-analyses suggesting that the HPA axis dysregulation might progress over time in patients with psychosis (Girshkin et al., 2014; Berger et al., 2016). However, previous studies have provided mixed findings on the role of epigenetic regulation of the *NR3C1* in the pathophysiology of psychotic disorders. Although Iftimovici et al. (2020) revealed that higher expression of the *NR3C1* gene might predict transition to overt psychosis in patients at clinical high risk, significant differences in *NR3C1* exon 1F methylation have not been confirmed (Schür et al., 2018; Liu et al., 2020). However, studies addressing methylation of *NR3C1* did not stratify the patients according to stage of illness. Importantly, the CpG sites that appeared to be differentially methylated in patients with FEP and SCZ-AR from our sample (especially CpG2, CpG4, and CpG8) are the binding sites for several transcription factors expressed in the brain and peripheral blood. Some of them, including EGR1, KLF5, SP1, SP4, and TFDP1, have been associated with the pathophysiology of schizophrenia (Katsel et al., 2008; Yanagi et al., 2008; Fusté et al., 2013; Duclot and Kabbaj, 2017). It is also important to note that in the majority of previous studies, only some CpG sites have been associated with various disease outcomes or psychosocial stress (for review, see Daskalakis and Yehuda, 2014). Some mechanistic insights into these associations originate from animal model studies. For instance, Bockmühl et al. (2015) found that early-life stress programs the *NR3C1* expression by site-specific methylation of the specific *NR3C1* region called “the CGI shore” in hypothalamic neurons that produce corticotropin-releasing hormone.

Another important finding from our study is that a history of any ACEs was associated with lower methylation of the *NR3C1* component 2 in the whole sample. Importantly, we found no association between recent or lifetime stressors and *NR3C1* methylation. Recent studies have shown that various environmental exposures, including early-life stress, can impact expression of the *NR3C1* gene, leading to long-term alterations of stress response and feedback regulation of the HPA axis (McGowan et al., 2009; Begum et al., 2013; Jiang et al., 2019). Although several previous studies have demonstrated higher levels of *NR3C1* methylation in individuals exposed to various ACEs (Nöthling et al., 2019), the opposite findings have also been reported. Schechter et al. (2015) found that parenting stress negatively correlates with the levels of *NR3C1* methylation in children. Another study demonstrated lower *NR3C1* exon 1F methylation in patients with generalized anxiety disorder with a history of ACEs (Wang et al., 2017). Lower methylation of the *NR3C1* has also been found in adults who lost their only child (Qi et al., 2020). Differences in previous studies might originate from assessment of various CpG sites in the *NR3C1* gene, heterogeneity of self-reports used for recording ACEs, and inclusion of various clinical populations.

Early-life stress may also impact methylation of other genes related to the pathophysiology of various mental disorders, such as *BDNF*, *COMT*, *MAOA*, *FKBP5*, and *SLC6A4* (Jiang et al., 2019). However, it remains unknown whether early-life stress is directly associated with altered DNA methylation. Indeed, several mediating and moderating factors are taken into consideration, including genetic variation, socioeconomic status, social support, individual resilience, or coping strategies (McEwen, 2016). For instance, Miller et al. (2020) found that posttraumatic stress disorder symptoms and resilience are associated with the *FKBP5* gene methylation in opposite directions. Clinical implications of these findings are yet to be established. There is evidence that epigenetic marks might predict treatment outcomes in various populations of patients with mental disorders (Goud Alladi et al., 2018). Additionally, it has been reported that psychopharmacological treatment may impact DNA methylation (Boks et al., 2012). However, it remains unknown whether stress-related epigenetic modifications might predict treatment response or serve as treatment targets.

Our study also demonstrated that methylation of the *NR3C1* gene might be related to worse performance of attention and immediate memory as well as lower general functioning in patients with psychosis but not in other groups of participants. However, a cross-sectional study design does not allow to make conclusions on the direction of causality. Nevertheless, this finding is in line with several observations that chronic exposure to glucocorticoids, associated with glucocorticoid resistance, leads to decreased hippocampal integrity (for review, see Conrad, 2008). Moreover, increased methylation of the *NR3C1* gene has been associated with decreased hippocampal connectivity (Palma-Gudiel et al., 2018). To our knowledge, the relationship between *NR3C1* methylation and cognition in patients with schizophrenia-spectrum disorders has not been tested so far. Impaired cognitive performance represents one of the key clinical characteristics of schizophrenia. According to some studies, cognitive decline tends to progress over time in this population (Vita et al., 2012; Zanelli et al., 2019). Increase in the level of *NR3C1* methylation and its negative correlation with cognition in patients with psychosis from our sample might be one of the mechanisms underlying this observation.

There are some important limitations of this study that need to be discussed. Firstly, our sample size was not large, especially regarding the size of specific subgroups of participants. Secondly, there were some significant between-group differences with respect to potential confounding factors, including age, BMI, and cigarette smoking rates. However, some of them, including BMI and cigarette smoking, were not significantly correlated with the levels of *NR3C1* methylation in the whole sample. In turn, the effects of age and the dosage of antipsychotics were not significant in the ANCOVA. Moreover, we obtained similar results when the analysis was limited to age-matched subgroups of participants. At this point, it should be noted that the CPZeq might be insufficient to address medication effects. Another limitation is that we did not record a number of psychotic exacerbations in SCZ-AR patients. Therefore, conclusions on the association between illness progression and *NR3C1* methylation should be made with caution. It is also important to note that we did not assess expression of the *NR3C1* gene, and thus the functional impact of differentially methylated CpG sites remains unknown. Moreover, the analysis of cortisol levels was based on single measurements. Finally, a cross-sectional study design does not provide insights into causal associations.

In summary, results of this study indicate that patients with schizophrenia-spectrum disorders show altered levels of *NR3C1* methylation that are significantly lower at early stages after the onset of psychosis and significantly higher after subsequent illness exacerbations. Increase in the level of *NR3C1* methylation might account for cognitive decline observed in schizophrenia. In turn, a history of ACEs might be associated with lower *NR3C1* methylation, and this observation is not specific to patients with psychosis. Longitudinal studies of patients with psychosis are needed to provide insights into causal associations between epigenetic regulation of the *NR3C1* gene, psychotic disorders, and cognitive decline.

## Supplementary Material

pyaa094_suppl_Supplementary_Materials
