# Corrigendum to: Clinical Correlates of the NR3C1 Gene Methylation at Various Stages of Psychosis

**DOI:** 10.1093/ijnp/pyab048

**Published:** 2021-08-04

**Authors:** Błażej Misiak, Jerzy Samochowiec, Anna Konopka, Barbara Gawrońska-Szklarz, Jan Aleksander Beszłej, Elżbieta Szmida, Paweł Karpiński

**Affiliations:** 1 Department of Psychiatry, Wroclaw Medical University, Wroclaw, Poland; 2 Department of Psychiatry, Pomeranian Medical University, Szczecin, Poland; 3 Department of Pharmacokinetics and Therapeutic Drug Monitoring, Pomeranian Medical University, Szczecin, Poland; 4 Independent Clinical Psychology Unit, Department of Psychiatry, Pomeranian Medical University, Szczecin, Poland; 5 Department of Genetics, Wroclaw Medical University, Wroclaw, Poland; 6 Laboratory of Genomics & Bioinformatics, Institute of Immunology and Experimental Therapy, Polish Academy of Sciences, Wroclaw, Poland

Misiak, B., Samochowiec, J., Konopka, A., Gawrońska-Szklarz, B., Aleksander Beszłej, J., Szmida, E., and Karpiński, P. (2021) Clinical Correlates of the NR3C1 Gene Methylation at Various Stages of Psychosis. *International Journal of Neuropsychopharmacology.*

In the originally published version of this manuscript, Dr Paweł Karpiński’s affiliation was incomplete. The correct affiliation is as follows: Department of Genetics, Wroclaw Medical University, Wroclaw, Poland and Laboratory of Genomics & Bioinformatics, Institute of Immunology and Experimental Therapy, Polish Academy of Sciences, Wroclaw, Poland. This has now been corrected online.

