# Supplementary material for: Clinical Correlates of the NR3C1 Gene Methylation at Various Stages of Psychosis
Source: Int J Neuropsychopharmacol. 2020 Dec 7;24(4):322–32. doi: 10.1093/ijnp/pyaa094 (PMC8059494; doi:10.1093/ijnp/pyaa094)
Supplement: pyaa094_suppl_Supplementary_Materials [file pyaa094_suppl_supplementary_materials.docx]

**Supplementary Appendix**

**Supplementary Figure 1.** The scree plot for the principal components analysis. A. whole sample; B. the sample with subgroups of participants matched for age (FEP, n = 25, 31.3 ± 5.6 years; SCZ-AR, n = 19, 34.9 ± 4.9 years; FHR-P, n = 19, 34.2 ± 5.4 years; HCs, n = 37, 34.6 ± 4.7 years; p = 0.063). The age-matched subgroups were extracted by limiting the analysis to participants aged 25 – 43 years.

**A.**


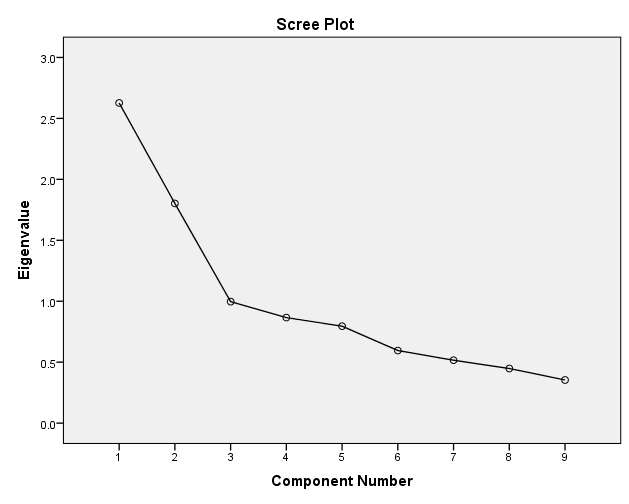


**B.**


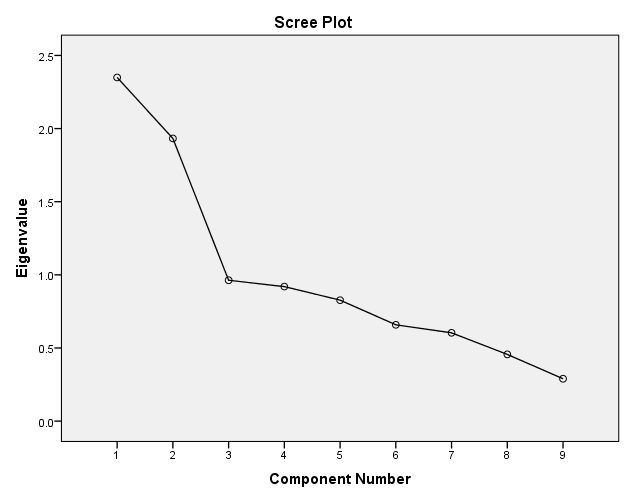


**Supplementary Figure 2.** Mean levels of the *NR3C1* methylation in the subgroups of participants matched for age (FEP, n = 25, 31.3 ± 5.6 years; SCZ-AR, n = 19, 34.9 ± 4.9 years; FHR-P, n = 19, 34.2 ± 5.4 years; HCs, n = 37, 34.6 ± 4.7 years; p = 0.063). The age-matched subgroups were extracted by limiting the analysis to participants aged 25 – 43 years. Error bars represent standard deviation. Abbreviations: FEP – first-episode psychosis; FHR-P – familial high risk of psychosis; HCs – healthy controls; SCZ-AR – acutely relapsed schizophrenia. *p < 0.05, **p < 0.01.

**
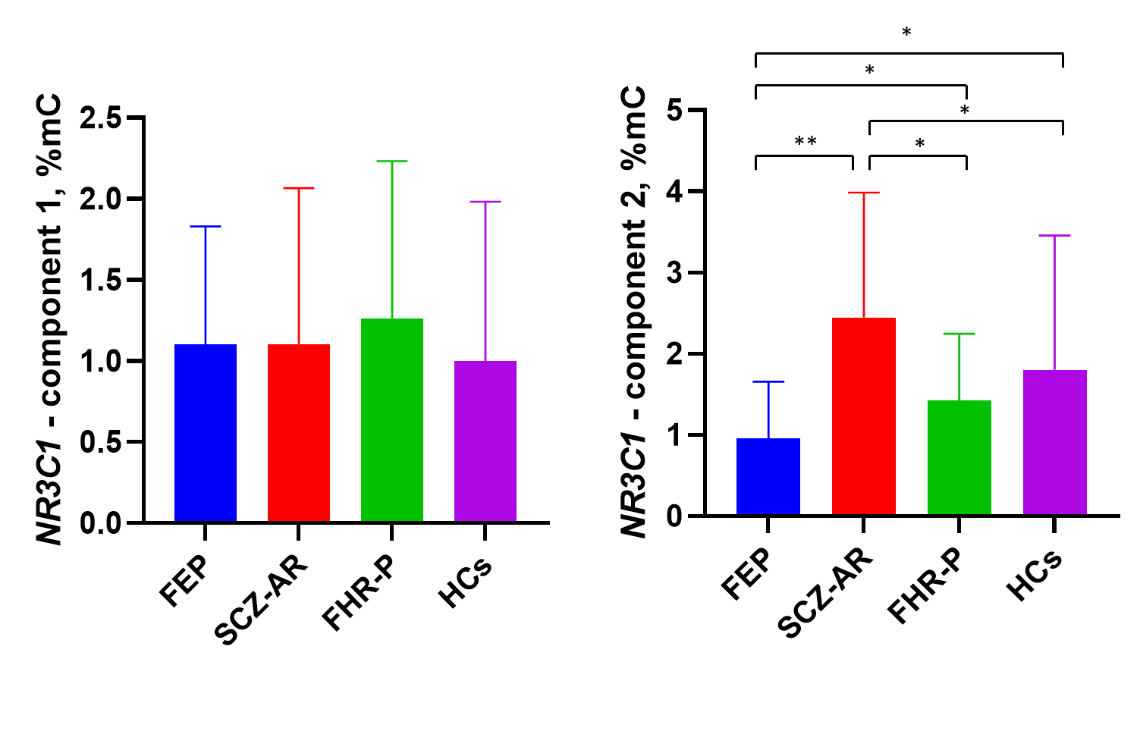
**

**Supplementary Table 1.** The pattern matrix of factor loadings

|  | Whole sample | | Sample with subgroups  matched for age* | |
| --- | --- | --- | --- | --- |
|  | Component 1 | Component 2 | Component 1 | Component 2 |
| CpG1 | 0.422 | -0.321 | 0.492 | -0.286 |
| CpG2 | -0.170 | 0.774 | -0.155 | 0.720 |
| CpG3 | 0.804 | -0.070 | 0.778 | -0.120 |
| CpG4 | -0.021 | 0.846 | 0.159 | 0.879 |
| CpG5 | 0.742 | 0.215 | 0.711 | 0.173 |
| CpG6 | 0.721 | -0.051 | 0.653 | -0.073 |
| CpG7 | 0.070 | 0.426 | -0.112 | 0.463 |
| CpG8 | 0.284 | 0.665 | 0.297 | 0.665 |
| CpG9 | 0.489 | 0.166 | 0.495 | 0.053 |

* The age-matched subgroups were extracted by limiting the analysis to participants aged 25 – 43 years.

**Supplementary Table 2.** Methylation levels (%mC) at single CpG sites of the *NR3C1* gene.

|  | 1. FEP | 2. SCZ-AR | 3. FHR-P | 4. HCs | p | Post-hoc analysis |
| --- | --- | --- | --- | --- | --- | --- |
| CpG1 | 0.325 ± 0.694 | 0.050 ± 0.181 | 0.487 ± 0.885 | 0.554 ± 1.249 | **0.007** | 2 < 4, 2 < 3 |
| CpG2 | 0.050 ± 0.221 | 2.156 ± 1.870 | 0.308 ± 1.104 | 1.304 ± 2.327 | **< 0.001** | 1 < 2, 1 < 4, 2 > 3, 2 > 4 |
| CpG3 | 1.600 ± 1.411 | 1.644 ± 1.667 | 1.897 ± 1.729 | 1.821 ± 2.037 | 0.921 | - |
| CpG4 | 1.725 ± 1.585 | 4.356 ± 2.551 | 2.744 ± 1.681 | 2.839 ± 2.947 | **< 0.001** | 1 < 2, 1 < 4, 2 > 3, 2 > 4 |
| CpG5 | 1.325 ± 1.328 | 1.289 ± 1.502 | 1.128 ± 1.341 | 0.607 ± 1.231 | **0.021** | 1 > 4 |
| CpG6 | 2.100 ± 1.795 | 1.911 ± 2.109 | 1.974 ± 1.709 | 1.982 ± 2.040 | 0.998 | - |
| CpG7 | 0.050 ± 0.316 | 0.178 ± 0.936 | 0.026 ± 0.160 | 0.054 ± 0.400 | 0.873 | - |
| CpG8 | 1.875 ± 1.667 | 3.044 ± 2.335 | 3.000 ± 1.919 | 2.429 ± 2.319 | **0.005** | 1 < 3, 1 < 2 |
| CpG9 | 0.050 ± 0.316 | 0.267 ± 0.863 | 0.179 ± 0.644 | 0.125 ± 0.662 | 0.491 | - |

Data expressed as mean ± SD

Significant differences (p < 0.05) were marked with bold characters

Abbreviations: FEP – first-episode psychosis; FHR-P – familial high risk of psychosis; HCs – healthy controls; SCZ-AR – acutely relapsed schizophrenia.

**Supplementary Table 3.** Association between potential confounding factors and methylation of the *NR3C1* gene.

|  | *NR3C1* component 1 | *NR3C1* component 2 |
| --- | --- | --- |
| Age | r = -0.047, p = 0.531 | **r = 0.209, p = 0.005** |
| Sex | Males vs. females: 1.10 ± 0.89 vs. 1.02 ± 0.93, p = 0.574 | Males vs. females: 1.73 ± 1.27 vs. 1.60 ± 1.40, p = 0.495 |
| Cigarette smoking | Non-smokers vs. smokers: 1.07 ± 0.91 vs. 1.10 ± 0.93, p = 0.625 | Non-smokers vs. smokers: 1.57 ± 1.37 vs. 1.77 ± 1.22, p = 0.269 |
| BMI | r = -0.055, p = 0.468 | r = -0.006, p = 0.938 |
| Illness duration | r = 0.109, p = 0.362 | **r = 0.444, p < 0.001** |
| CPZeq | r = 0.095, p = 0.409 | **r = 0.266, p = 0.020** |
| Cortisol | r = -0.041, p = 0.613 | r = -0.048, p = 0.555 |
| Somatic comorbidities | Present vs. absent: 1.29 ± 1.01 vs. 1.01 ± 0.89, p = 0.178 | Present vs. absent: 1.53 ± 1.05 vs. 1.68 ± 1.39, p = 0.902 |
| Non-psychiatric medications | Yes vs. no: 0.73 ± 0.90 vs. 1.10 ± 0.91, p = 0.061 | Yes vs. no: 1.74 ± 1.38 vs. 1.65 1.34, p = 0.762 |

Significant associations (p < 0.05) were marked with bold characters.

Abbreviations: CPZeq – chlorpromazine equivalent dosage, BMI – body mass index
